# Assessment of ChatGPT’s Compliance with ESC-Acute Coronary Syndrome Management Guidelines at 30-Day Intervals

**DOI:** 10.3390/life14101235

**Published:** 2024-09-27

**Authors:** Muhammet Geneş, Murat Çelik

**Affiliations:** 1 Cardiology Residency, Department of Cardiology, Sincan Training and Research Hospital, Sincan, Ankara 06930, Turkey; 2 Department of Cardiology, Gulhane Training and Research Hospital, Health Science University, Ankara 06000, Turkey; drcelik00@hotmail.com

**Keywords:** acute coronary syndromes, artificial intelligence, ChatGPT, ESC guidelines, clinical decision support, AI in healthcare

## Abstract

**Background:** Despite ongoing advancements in healthcare, acute coronary syndromes (ACS) remain a leading cause of morbidity and mortality. The 2023 European Society of Cardiology (ESC) guidelines have introduced significant improvements in ACS management. Concurrently, artificial intelligence (AI), particularly models like ChatGPT, is showing promise in supporting clinical decision-making and education. **Methods:** This study evaluates the performance of ChatGPT-v4 in adhering to ESC guidelines for ACS management over a 30-day interval. Based on ESC guidelines, a dataset of 100 questions was used to assess ChatGPT’s accuracy and consistency. The questions were divided into binary (true/false) and multiple-choice formats. The AI’s responses were initially evaluated and then re-evaluated after 30 days, using accuracy and consistency as primary metrics. **Results:** ChatGPT’s accuracy in answering ACS-related binary and multiple-choice questions was evaluated at baseline and after 30 days. For binary questions, accuracy was 84% initially and 86% after 30 days, with no significant change (*p* = 0.564). Cohen’s Kappa was 0.94, indicating excellent agreement. Multiple-choice question accuracy was 80% initially, improving to 84% after 30 days, also without significant change (*p* = 0.527). Cohen’s Kappa was 0.93, reflecting similarly high consistency. These results suggest stable AI performance with minor fluctuations. **Conclusions:** Despite variations in performance on binary and multiple-choice questions, ChatGPT shows significant promise as a clinical support tool in ACS management. However, it is crucial to consider limitations such as fluctuations and hallucinations, which could lead to severe issues in clinical applications.

## 1. Introduction

Even with ongoing advancements in healthcare and preventive strategies, heart disease and the various conditions grouped under acute coronary syndromes (ACSs) remain the leading causes of illness and death [1]. These syndromes include a spectrum of clinical scenarios characterized by new or changing symptoms and signs, which may or may not be evident on a standard ECG and could involve fluctuations in cardiac troponin (cTn) levels. The 2023 ESC (European Society of Cardiology) guidelines are crucial for addressing these clinical scenarios. These guidelines provide a detailed framework for the early assessment and ongoing management of adults presenting with these heart conditions [2,3,4].

Artificial intelligence (AI) is a domain of computer science that strives to create systems endowed with the capability to emulate cognitive functions characteristic of human beings [3]. These systems are designed to independently process information and perform tasks by gleaning knowledge from their digital surroundings. Within the AI spectrum, machine learning stands out by utilizing algorithms to derive insights and forecast outcomes based on data. Natural language processing (NLP) is another subset of AI designed to interpret and formulate conversation in a manner reminiscent of human interaction [4]. Historically, Alan Turing introduced the idea of using machines to generate knowledge from experience and make predictions based on that knowledge, laying the groundwork for AI, which was further developed by John McCarthy with the concept of “the science and engineering of creating intelligent machines.” Between the 1950s and 1970s, AI development focused on mimicking human cognitive functions, although its adoption in medicine was gradual. AI development slowed from the 1970s to the 2000s, but systems like the DXplain decision support system, developed in 1986, played a crucial role in providing medical knowledge. From 2000 to 2020, the use of AI in medicine accelerated, with systems like IBM Watson becoming instrumental in medical decision-making [5]. In cardiology, AI algorithms, particularly convolutional neural networks (CNNs) and other machine learning models, have revolutionized the interpretation of medical images such as cardiac MRI, CT scans, and echocardiograms. AI-driven solutions enhance the capabilities of healthcare providers, streamline processes, reduce diagnostic errors, and enable proactive interventions [6]. Lately, AI’s scope has broadened to encompass sectors, including healthcare for diagnostic purposes, the Internet of Things, and the creation of smart devices [4,7]. Representing a sophisticated echelon in language models, ChatGPT utilizes profound neural network methodologies to simulate NLP responses based on textual input. As a creation of Open AI, it integrates into the expansive generative pre-trained transformer (GPT) model lineage. It is acknowledged as one of the most comprehensive language models accessible to the public. By analyzing a vast array of textual information, ChatGPT skillfully discerns nuanced linguistic elements, thus equipping it to formulate relevant and nuanced dialogue across diverse contexts. While AI has been a mainstay in customer service and information management for some time, its application in healthcare and medical research is burgeoning. Including AI within these sectors is anticipated to boost operational efficacy and diagnostic accuracy significantly [8].

Incorporating AI into clinical practice will refine decision-making processes, enhance diagnostic precision, optimize therapeutic strategies, and improve patient outcomes. Our primary goal is to explore the potential of AI models to support clinicians who have a foundational understanding of acute coronary syndrome and require verification or recall of high-evidence recommendations (including timing, drug priorities, dosages, indications, and contraindications). Additionally, we aim to assess the learning and developmental capabilities of AI over a specific time period. This study contributes to the expanding literature on AI applications in cardiology by examining the alignment of advanced language models with cardiology guidelines.

## 2. Methods

**Study Design:** This research was designed to evaluate the performance of ChatGPT-4 in aligning with the standards of the 2023 ESC guidelines for the diagnosis, treatment, and management of ACS. The study aimed to assess the applicability of ChatGPT-4 as a decision-support tool for clinicians managing ACS patients, focusing on its accuracy, reliability, role as an educational tool for clinicians, and its capacity for learning and improvement over time. Ethical approval and informed consent were deemed unnecessary since the study did not involve human or animal participants. The research was conducted through systematic inquiries derived from the ESC’s ACS management guidelines. Ethical compliance was maintained throughout the study in accordance with the moral principles of the Declaration of Helsinki.

**Study Environment:** A dataset of 100 questions based on the 2023 ESC Clinical Practice Guidelines for ACS management was created to reflect the theoretical complexities of clinical decision-making. These questions, composed of text, tables, and figures containing high-level evidence recommendations like Class 1 and Class 3, were developed by two cardiology experts (M.G. and M.Ç), each preparing 50 questions (M.G. created multiple-choice questions, while M.Ç. prepared true/false questions) (Supplementary Tables S1 and S2). M.G. and M.Ç. mutually verified the datasets according to the ESC guidelines. All responses provided by ChatGPT were independently evaluated by M.G and M.Ç, and the results were statistically compared. Before initiating the question-and-answer sequence, the relevant guideline was not uploaded to any Chat-GPT environment, nor was there any redirection to ESC or academic websites. The questions had not been tested on the Chat-GPT platform beforehand.

The questions were divided into two categories for detailed evaluation: (1) true/false questions (a total of 50), designed to measure the accuracy of the AI in confirming or refuting statements based on the ESC guidelines for ACS management, and (2) multiple-choice questions (also a total of 50), designed to evaluate the AI’s ability to select the correct option among multiple choices following the same guidelines. The questions were presented to Chat-GPT on the same day, with the AI being restarted and the order of questions and options shuffled, ensuring that consistency was maintained as the responses did not change. Additionally, it was confirmed that Chat-GPT had not received any significant updates over the 30 days.

These questions were strategically selected to quantitatively evaluate the accuracy of the AI, with responses assessed as correct or incorrect. The initial and subsequent performance of ChatGPT was evaluated 30 days apart, a period chosen at random to introduce varying levels of complexity. This interval was selected to monitor fluctuations in AI responses over time and thus assess its learning capability. The period was deemed optimal for observing performance changes in the AI without requiring a significant update to the GPT model. The questions covered various relevant topics, including diagnostic criteria, therapeutic strategies, and other critical issues.

## 3. AI Tool: ChatGPT-v4 and Analysis of Results

**Study Design and Evaluation:** In this study, ChatGPT-4, developed by OpenAI, was employed from 1 March to 30 March 2024, as an NLP tool to assist complex decision-making processes. The AI’s responses were assessed using the Likert scale, a commonly used method in research for evaluating attitudes and perceptions. This study utilized a six-point Likert scale to measure the accuracy of each response: 1 for completely false, 2 for mostly false, 3 for equally true and false, 4 for more true than false, 5 for almost entirely true, and 6 for completely true. Additionally, the completeness of each response was evaluated on a three-point scale: 1 for Inadequate (addressing some aspects but missing or incomplete in crucial areas), 2 for Adequate (covering all necessary elements with sufficient information), and 3 for Comprehensive (providing thorough coverage with additional context beyond expectations). In line with these criteria, a total of 50 true/false and 50 multiple-choice questions were designed. Following each response, whether correct or incorrect, ChatGPT-4 attempted to provide explanatory information, similar to its approach with multiple-choice questions, allowing for further evaluation based on the Likert scale. The responses were reviewed by two experienced cardiologists (M.G. and M.Ç.) for accuracy, relevance, and adherence to ESC guidelines. The analysis primarily focused on three aspects: Accuracy, measured by the percentage of correct responses; Consistency, reflecting the AI’s ability to provide stable answers to similar questions; and Comprehensiveness, assessing the depth and scope of responses in covering the key elements of the ESC guidelines. A vital aspect of the study involved re-evaluating answers initially deemed inaccurate by ChatGPT (those scoring below three on the accuracy scale) to determine the effect of time on the AI’s performance. Accordingly, after a 30-day interval, the same questions were presented to ChatGPT again, and the updated responses were re-evaluated and scored.

## 4. Statistical Analysis

The data gathered from the study were aggregated and examined using descriptive statistical approaches, including calculating the median—the midpoint of the ordered data set—and the mean, reflecting the average value of the collected data. To assess the temporal stability of ChatGPT’s answers regarding ACS, the chi-square test was used to determine the variance in correct versus incorrect responses over assessments carried out initially and 30-day later. Before making group comparisons, the assumption of normal distribution was evaluated with the Shapiro–Wilk test. Non-parametric tests were used because the data did not exhibit normal distribution in the groups (Shapiro–Wilk *p* < 0.05). The Mann–Whitney U test was used to compare two distinct groups, and the Kruskal–Wallis test was applied for analyses involving more than two groups. For sequential assessment of the responses, the Wilcoxon signed-rank test was conducted, which is pertinent for comparing paired samples or the same sample across different times to determine if the average ranks are significantly different. Bivariate tests were performed, with a *p* < 0.05 set as the threshold for statistical significance. Data analysis was conducted using SPSS software, version 26, provided by IBM Corporation, Armonk, NY, USA.

## 5. Results

### 5.1. Binary Questions

**Figure 1:**
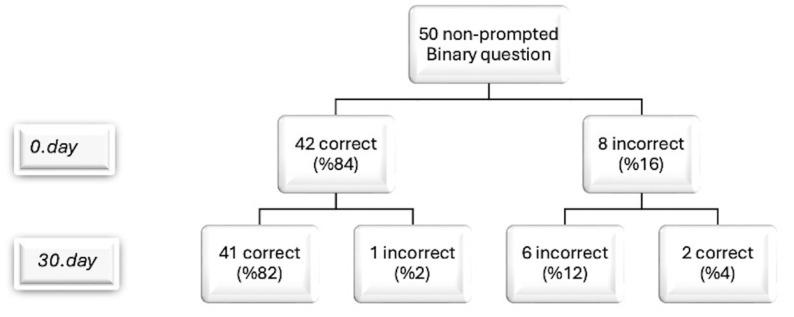
Flowchart showing the variability in ChatGPT-V4’s responses to the same binary questions posed by observers on days 0 and 30.

In analyzing responses to binary questions related to ACSs, the accuracy of ChatGPT’s answers was compared at baseline and after 30 days (as shown in Table 1). At the outset, 84% of responses (42) were aligned with the guidelines. Upon re-evaluation after 30 days, no improvement was observed (Figure 1). Two non-conforming responses, representing 25% of incorrect answers and 4% of the total, were corrected to align with the guidelines, indicating a positive trend. However, one initially correct response shifted to non-conformity, leading to a 2.4% decrease in accurate responses. Overall, despite some corrections, the net result was seven non-conforming outcomes. These findings suggest a nuanced picture of AI performance stability and adherence to guidelines, with a 4% improvement in previous errors counterbalanced by a 2% regression in initially correct responses. The initial success rate of ChatGPT, 0.84 (±0.37), and the success rate after 30 days, 0.86 (±0.35), were not found to be statistically significantly different (*p* = 0.564). The Cohen’s Kappa coefficient for binary questions was 0.94, indicating an excellent level of agreement, similar to the high concordance observed in binary questions (*p* < 0.001).

### 5.2. Multiple-Choice Questions

**Figure 2:**
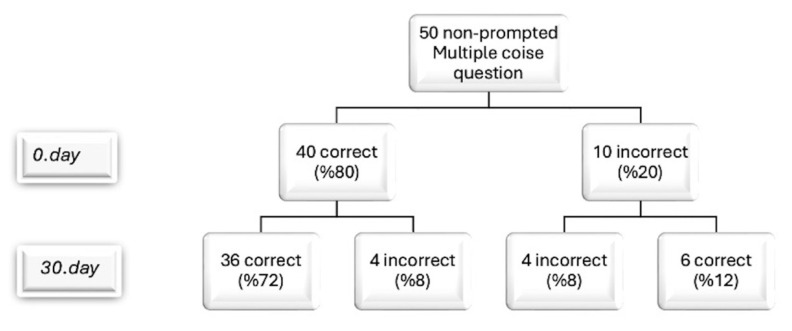
Flowchart showing the variability in ChatGPT-V4’s responses to the same multiple-choice questions posed by observers on days 0 and 30.

The precision of ChatGPT’s answers in the multiple-choice test was evaluated initially and after a 30-day interval (Figure 2). Initially, 80% of responses (40) were consistent with the guidelines. At the 30-day reassessment, four of the ten previously inconsistent responses (40% of incorrect and 8% of total responses) showed no improvement. However, six non-conforming responses (60% of incorrect and 12% of total responses) were corrected to align with the guidelines, indicating a positive trend. Notably, 4 of the initially correct 40 responses (10% of correct and 8% of total responses) shifted to non-conformity, resulting in 8 non-conforming outcomes overall. No statistically significant difference was found between the initial success rate of ChatGPT, 0.80 (±0.40), and the correct success rate after 30 days, 0.84 (±0.37) (*p* = 0.527). The Cohen’s Kappa coefficient for multiple-choice questions was 0.93, indicating an excellent level of agreement, similar to the high concordance observed in binary questions (*p* < 0.001).

## 6. Discussion

ACS is characterized by a sudden reduction in blood flow to the heart, with over 7 million people diagnosed annually worldwide, making it a leading cause of mortality [2]. Clinicians evaluating potential ACS patients must act with a high degree of suspicion and attentiveness. Timely diagnosis and treatment of ACS are critical for reducing mortality and morbidity. This study is one of the first to evaluate ChatGPT’s ability to generate responses consistent with ESC guidelines for ACS. Our findings suggest that ChatGPT has the potential to be a valuable tool for clinicians in need of quick and accurate information on ACS management. However, the lack of evidence-based support in some of ChatGPT’s responses highlights critical areas that need consideration in the development of NLP tools.

There are limited studies assessing the accuracy of ChatGPT’s responses to clinical questions based on guidelines. Existing research indicates that these systems are partially successful in generating appropriate medical responses [8,9,10,11]. Therefore, AI robots may have significant potential in clinical decision-making. Our study observed that ChatGPT provided correct responses to many clinical questions related to ACS management according to the 2023 ESC guidelines. ChatGPT initially answered 80% of the 50 multiple-choice questions and 84% of the binary questions related to ACS correctly. After 30 days, the accuracy rate increased to 85%, but a 15% error rate persisted. While this accuracy rate suggests ChatGPT’s potential as a valuable tool, the existing error rate raises concerns about guideline adherence and highlights worrisome inconsistencies. It is known that while ChatGPT can generate responses to scientific questions, some of the data and references it produces may be a blend of accurate and completely fabricated information. It is important to remember that AI systems like ChatGPT, despite their vast databases and powerful computing capacities, create text based on statistical calculation algorithms, attempting to identify problems rather than truly understanding them [12,13]. The probability maximization used in response generation can lead to inconsistent or repetitive, cyclical texts and content described as “hallucinations,” meaning internally or externally nonsensical content not faithful to the source. Hallucinative texts may appear correct but do not reflect reality; detecting this at first glance can be challenging [14]. Hallucinations in AI pose significant concerns, especially in applications directly impacting human lives like medical practice. This situation necessitates caution when using AI models in medical applications. To achieve reliable results, these models should be trained not with vast and contextually diverse datasets but with accurate datasets from reliable sources, supplemented with continuous user feedback and evaluations. This approach could reduce AI hallucinations alongside large data drifts and temporal degradation issues.

Unlike previous ones, a distinctive aspect of our study was the re-evaluation of the same questions at 30-day intervals. The purpose of this interval was to assess the stability and improvement of ChatGPT’s performance over time. It was observed that 8% of ChatGPT’s initially correct answers to the 50 multiple-choice questions became incorrect after 30 days. However, 60% of the initially incorrect answers were corrected, indicating a positive change. Similarly, for the 50 binary questions, 82% of the initially correct answers remained accurate, though one correct answer shifted to incorrect. Additionally, 25% of the initially incorrect answers were corrected. This analysis highlights fluctuating performance in maintaining correct responses over time, with improvements in initially incorrect answers balanced by a slight decline in correct ones. The inconsistency and variability, termed AI performance fluctuation, stem from several factors. Chief among these is the heavy reliance on AI models like ChatGPT on the data on which they are trained. The primary texts used in training NLP models do not fully capture the complexity of real-world patient scenarios. As a result, AI systems lack clinical intuition and experience. The context-dependent and complex nature of medical data makes it challenging for AI to interpret them accurately, potentially leading to a decline in performance over time. Additionally, biases in training data can influence AI outputs, leading to incorrect or incomplete responses, particularly for patient groups underrepresented in the training data. Moreover, due to algorithmic limitations, AI models may not always correctly understand the context or make errors when generalizing from the training data, resulting in different outcomes for similar questions. Another factor influencing response variability is the frequency of AI model updates. Without regular updates, the progression of medical knowledge and the introduction of new guidelines may cause the model to provide less accurate responses over time [6,15].

An important concept to consider is prompt engineering. AI language models generate responses based on the prompts or the questions/instructions provided by the user. Variability in answers can be attributed to how the question is phrased, the clarity of the context, and the algorithms involved in response generation. Clear and specific questions tend to produce more consistent answers, while general or vague questions may lead to more variable responses.

In our research, while AI tended to maintain the majority of its initial accuracy levels, it remained constant in correcting initial errors. This suggests that there may be stagnation in the current learning capacity of the model, likely stemming from the AI’s internal mechanisms and periodic updates. ChatGPT received its last major update in October 2023. While AI can be used as a supportive tool by clinical researchers and doctors, its capacity to replace human tasks in complex data collection, analysis, and validation processes remains uncertain. Therefore, further research is necessary for integrating AI into clinical medicine, and clinicians must approach AI tools with some skepticism. The initial success of AI in binary and multiple-choice questions also highlights the need for continuous updates, as responses risk becoming outdated over time. In conclusion, although this AI model achieves good accuracy, it is fundamentally a research version of a conversational tool. It was not designed as a medical tool; hence, while it holds great potential in the medical field, there is still a long way to go.

## 7. Limitations

This study has several limitations. First, the relatively small sample size and the focus of the questions primarily on text interpretation and information processing may limit the generalizability of the results. Second, the 30-day evaluation period may not fully capture the learning capacity of the AI, and the observed changes could reflect natural variability rather than actual improvements. Testing AI tools after significant updates or over longer intervals could be beneficial to better understanding these technologies’ development. Additionally, as indicated by the p-values, the lack of significant changes in accuracy and completeness scores suggests that the observed differences may result from random fluctuations rather than systematic improvements or deteriorations in AI performance. Future studies could address these limitations by incorporating specific clinical scenarios to evaluate AI’s data interpretation abilities or directly comparing human and AI performance.

## 8. Conclusions

Alongside the immense potential of AI, it is crucial to fully understand its limitations, such as fluctuations and hallucinations, which could cause significant harm in clinical applications. To fully realize the potentially revolutionary impact of AI in medicine and healthcare, ongoing research with advanced models is of great importance. Future studies should aim to enhance AI’s comprehensive understanding of biological and medical sciences, improve the accuracy of its algorithms in diagnosis and treatment planning, and address its shortcomings. Additionally, the issue of unauthorized use should be discussed, and ethical guidelines, limitations, and accreditations should be developed by competent authorities to prevent harmful usage and to protect patient confidentiality and privacy.

## Figures and Tables

**Table 1:** Comparison of the mean scores of accuracy and completeness for binary and multiple-choice formats at two different time points: the initial assessment and after 30 days.

Variable		İnitial	30th Day	p *
Multiple	Success Rate	0.80 (±0.40)	0.84 (±0.37)	0.527
Binary	Success Rate	0.84 (±0.37)	0.86 (±0.35)	0.564
**p** +		0.604	0.781	
Multiple	Accuracy	5.06 (±1.55)	5.20 (±1.32)	0.490
Binary	Accuracy	5.30 (±1.39)	5.36 (±1.35)	0.785
**p** +		0.387	0.275	
Multiple	Completeness	2.56 (±0.92)	2.56 (±0.83)	0.971
Binary	Completeness	2.54 (±0.70)	2.52 (±0.70)	0.655
**p** +		0.915	0.880	

* Wilcoxon signed-rank test,+ Mann Whitney U Test.

## Data Availability

The data supporting this study’s findings are available from the corresponding author upon reasonable request.

## Supplementary Materials

The following supporting information can be downloaded at https://www.mdpi.com/article/10.3390/life14101235/s1: Table S1. Multiple-choice questions. Table S2. Binary questions.
