# Supplementary material for: Assessment of ChatGPT’s Compliance with ESC-Acute Coronary Syndrome Management Guidelines at 30-Day Intervals
Source: Life (Basel). 2024 Sep 27;14(10):1235. doi: 10.3390/life14101235 (PMC11508737; doi:10.3390/life14101235)
Supplement: Supplementary file 1 [file life-14-01235-s001.zip › Supplement Table S1.pdf]

Supplement Table S1. Binary Questions

Question	Initial Answer	30th day Answer
**Q1)** Patients with acute coronary syndrome must have an electrocardiographic change for diagnosis	**T**	**T**
**Q2)** Patients with acute coronary syndrome must have elevated troponin levels for diagnosis	**T**	**T**
**Q3)** Acute myocardial infarction is defined as cardiomyocyte necrosis in the setting of acute myocardial ischemia	**T**	**T**
**Q4)** The emergency percutaneous coronary intervention performed as soon as possible following the failure of fibrinolytic therapy is referred to as early percutaneous coronary intervention.	**T**	**T**
**Q5)** The rescue percutaneous coronary intervention strategy performed between 2 to 24 hours after successful fibrinolysis, if indicated, is the percutaneous coronary intervention of the infarct-related artery	**T**	**T**
**Q6)** Early coronary angiography (within <24 hours of ACS diagnosis) and, if indicated, percutaneous coronary intervention/coronary artery bypass grafting of the infarct-related artery are referred to as an early invasive strategy	**F**	**F**
**Q7)** If patients presenting with ACS stop DAPT to undergo coronary artery bypass grafting, it is recommended they resume DAPT after surgery for at least 6 months	**F**	**F**
**Q8)** De-escalation of antiplatelet therapy in the first 60 days after an ACS event is not recommended	**T**	**T**
**Q9)** Evaluation of neurological prognosis (no earlier than 48 h after admission) is recommended in all comatose survivors after cardiac arrest	**F**	**F**
**Q10)** In patients with spontaneous coronary artery dissection, PCI is recommended only for patients with symptoms and signs of ongoing myocardial ischemia, a large area of myocardium in jeopardy, and reduced antegrade flow	**T**	**T**
**Q11)** It is recommended that PCI of the non-IRA is based on angiographic severity**.**	**F**	**F**
**Q12)** Invasive epicardial functional assessment of non-culprit segments of the IRA is recommended during the index procedure	**T**	**T**
**Q13)** Implantation of a permanent pacemaker is recommended when high-degree AV block does not resolve within a waiting period of at least 7 days after MI	**T**	**T**
**Q14)** It is recommended to base the choice of long-term glucose-lowering treatment on the presence of comorbidities, including heart failure, chronic kidney disease, and obesity	**T**	**T**
**Q15)** For frail older patients with comorbidities, a holistic approach is recommended to individualize interventional and pharmacological treatments after careful evaluation of the risks and benefits.	**T**	**T**
**Q16)** An invasive strategy is recommended in cancer patients presenting with high-risk ACS with expected survival ≥3 months	**F**	**F**
**Q17)** A temporary interruption of cancer therapy is recommended in patients in whom the cancer therapy is suspected to be a contributing cause of ACS	**T**	**T**
**Q18)** Aspirin is not recommended in cancer patients with a platelet count <30.000/ml	**T**	**T**
**Q19)** Clopidogrel is not recommended in cancer patients with a platelet count <30.000/ml	**T**	**T**
**Q20)** In ACS patients with cancer and <70.000/ml platelet count, prasugrel or ticagrelor are not recommended	**T**	**T**
**Q21)** It is recommended to intensify lipid-lowering therapy during the index ACS hospitalization for patients who were on lipid-lowering therapy before admission	**T**	**T**
**Q22)** Routine immediate angiography after resuscitated cardiac arrest is recommended in hemodynamically stable patients without persistent ST-segment elevation (or equivalents)	**T**	**T**
**Q23)** Temperature control (i.e. continuous monitoring of core temperature and active prevention of fever [i.e.>37,7°C]) is recommended after either out-of-hospital or in-hospital cardiac arrest for adults who remain unresponsive after return of spontaneous circulation	**T**	**T**
**Q24)** Complete revascularization is recommended either during the index PCI procedure or within 45 days	**T**	**T**
**Q25)** Twelve-lead ECG recording and interpretation is recommended as soon as possible at the point of FMC, with a target of < 30 min	**T**	**F***
**Q26)** It is recommended to use an ESC algorithmic approach with serial hs-cTn measurements (0 h/1 h or 0 h/2 h) to rule in and rule out NSTEMI	**T**	**T**
**Q27)** Routine, early CCTA in patients with suspected ACS is recommended	**F**	**F**
**Q28)** Routine oxygen is not recommended in patients without hypoxemia (SaO2 >90%)	**T**	**T**
**Q29)** A PPCI strategy is recommended over fibrinolysis if the anticipated time from diagnosis to PCI is < 90 min	**T**	**T**

**Q30)** If timely PPCI (<120 min) cannot be performed in patients with a working diagnosis of STEMI, fibrinolytic therapy is recommended within 12 h of symptom onset in patients without contraindications	**T**	**T**
**Q31)** Routine PCI of an occluded IRA is recommended in STEMI patients presenting >48 h after symptom onset and without persistent symptoms	**T**	**T**
**Q32)** Prasugrel is recommended in P2Y12 receptor inhibitor-naïve patients proceeding to PCI (60 mg LD, 10 mg o.d. MD, 5 mg o.d. MD for patients aged ≥75 years or with a body weight	**F**	**T***
**Q33)** Ticagrelor is recommended irrespective of the treatment strategy (invasive or conservative) (180 mg LD, 90 mg b.i.d. MD)	**T**	**T**
**Q34)** Pre-treatment with a GP IIb/IIIa receptor antagonist is recommended	**T**	**T**
**Q35)** Routine use of a UFH bolus (weight-adjusted i.v. bolus during PCI of 70–100 IU/kg) is recommended in patients undergoing PCI	**T**	**T**
**Q36)** Fondaparinux is recommended in patients with STEMI undergoing PPCI	**T**	**T**
**Q37)** The use of ticagrelor or prasugrel as part of triple antithrombotic therapy is recommended	**T**	**T**
**Q38)** The routine use of an IABP in ACS patients with CS and without mechanical complications is recommended	**T**	**T**
**Q39)** Routine echocardiography is recommended during hospitalization to assess regional and global LV function, detect mechanical complications, and exclude LV thrombus	**T**	**T**
**Q40)** It is recommended that high-risk patients (including all STEMI patients and very high-risk NSTE-ACS patients) have ECG monitoring for a minimum of 24 h	**T**	**T**
**Q41)** The routine use of thrombus aspiration is not recommended	**T**	**T**
**Q42)** Drug-eluting stents are recommended in preference to bare metal stents in all cases	**T**	**T**
**Q43)** In patients with a working diagnosis of MINOCA, CMR imaging is recommended after invasive angiography if the final diagnosis is not clear	**T**	**T**
**Q44)** It is recommended to aim to achieve an LDL-C level of <1.4 mmol/L (<55 mg/dL) and to reduce LDL-C by ≥50% from baseline	**T**	**T**
**Q45)** If the LDL-C goal is not achieved despite maximally tolerated statin therapy after 4–6 weeks, the addition of ezetimibe is recommended	**T**	**T**
**Q46)** Beta-blockers are recommended in ACS patients with LVEF >40% regardless of HF symptoms	**T**	**T**
**Q47)** Influenza vaccination is recommended for all ACS patients	**T**	**T**
**Q48)** Routine pre-treatment with a P2Y12 receptor inhibitor in NSTE-ACS patients in whom coronary anatomy is not known and early invasive management (<24 h) is planned is not recommended	**F**	**T***
**Q49)** Treatment of asymptomatic and hemodynamically irrelevant ventricular arrhythmias with anti-arrhythmic drugs is recommended	**T**	**T**
**Q50)** Pacing is not recommended if high-degree AV block resolves after revascularization or spontaneously	**T**	**T**

*: Questions initially answered incorrectly by ChatGPT-4 but subsequently corrected when asked again on the 30th day.
