# Supplementary material for: Assessment of ChatGPT’s Compliance with ESC-Acute Coronary Syndrome Management Guidelines at 30-Day Intervals
Source: Life (Basel). 2024 Sep 27;14(10):1235. doi: 10.3390/life14101235 (PMC11508737; doi:10.3390/life14101235)
Supplement: Supplementary file 1 [file life-14-01235-s001.zip › Supplement Table S2.pdf]

Supplement Table S2. Multiple choice questions

Question	Initial Answer	30th day Answer
**Q1)** Which of the following statements about a patient with multiple cardiac risk factors and angina-like chest pain lasting 20 minutes is incorrect?	T	T
**Q2)** In patients presenting with chest pain to the emergency room (ER), according to guideline recommendations, how soon should an electrocardiogram (ECG) be performed upon arrival?	F	T*
**Q3)** What is the recommended loading dose of aspirin for patients with acute coronary syndrome according to guidelines?	T	T
**Q4)** What is the recommended maintenance dose of aspirin for patients with acute coronary syndrome according to guidelines?	T	T
**Q5)** What is the recommended loading dose of ticagrelor for patients with acute coronary syndrome according to guidelines?	T	T
**Q6)** What is the recommended maintenance dose of ticagrelor for patients with acute coronary syndrome according to guidelines?	T	T
**Q7)** What is the recommended loading dose of prasugrel for patients with acute coronary syndrome according to guidelines?	T	T
**Q8)** What is the recommended maintenance dose of prasugrel for patients with acute coronary syndrome according to guidelines?	F	T*
**Q9)** For patients who stop dual antiplatelet therapy (DAPT) due to coronary artery bypass grafting (CABG) after presenting with acute coronary syndrome (ACS), how many months is it recommended to continue DAPT post-surgery?	T	T
**Q10)** How many days after an ACS event can de-escalation of antiplatelet therapy be performed according to guideline recommendations?	T	T
**Q11)** Which of the following is one of the criteria for an emergency invasive strategy for non-ST elevation acute coronary syndrome?	T	T
**Q12)** Based on the available data, which of the following statements regarding revascularisation recommendations in the setting of ACS is correct?	T	T
**Q13)** Which of the following statements are correct regarding prasugrel use?	T	T
**Q14)** Which of the following is not a criterion for an early invasive strategy in non-ST elevation acute coronary syndrome?	T	T
**Q15)** In patients diagnosed with ST-elevation myocardial infarction (STEMI), fibrinolytic therapy is recommended if primary percutaneous coronary intervention (PPCI) cannot be performed within what timeframe?	F	F
**Q16)** For patients presenting with acute myocardial infarction (MI), what are the desirable plasma glucose targets?	T	T
**Q17)** Under which circumstances are adjunctive GP IIb/IIIa inhibitors recommended in addition to dual antiplatelet therapy (DAPT) and heparin in the acute ST-segment elevation myocardial infarction (STEMI) setting?	T	T
**Q18)** Which of the following statements is correct regarding acute ST-segment elevation myocardial infarction (STEMI) qualifying for reperfusion therapy?	F	F
**Q19)** Which of the following is correct for routine early PCI strategy after fibrinolysis?	T	T
**Q20)** When is a bare metal stent preferred in patients?	T	T
**Q21)** Which of the following information about spontaneous coronary dissection is correct?	T	T
**Q22)** Which of the following is incorrect regarding the management of acute coronary syndrome during hospitalization?	F	F
**Q23)** Which is incorrect about the approach to patients with cardiogenic shock complicating acute coronary syndrome?	T	T
**Q24)** Which one is incorrect about out-of-hospital cardiac arrest in acute coronary syndrome?	T	T
**Q25)** Which one is incorrect about the recommendations for fibrinolytic therapy in patients with ACS?	T	T
**Q26)** How long after acute coronary syndrome (ACS) should antiplatelet therapy be discontinued in patients treated with an oral anticoagulant (OAC)?	F	T*
**Q27)** Which of the following is correct regarding antiplatelet and anticoagulant treatment recommendations in acute coronary syndrome?	T	F*
**Q28)** What is the weight-adjusted routine UFH bolus dose in patients undergoing PCI for acute coronary syndrome?	T	T
**Q29)** What is the target LDL-c level in patients with acute coronary syndrome?	T	T
**Q30)** Which of the following is incorrect regarding recommendations for a healthy lifestyle in ACS patients?	T	T
**Q31)** In patients with acute coronary syndrome, if the LDL-C target is not reached after 4-6 weeks despite maximally tolerated statin therapy, which agent is recommended to be added first?	T	T
**Q32)** After how long following acute coronary syndrome (ACS) is reassessment of left ventricular ejection fraction (LVEF) recommended in patients with pre-discharge LVEF ≤40% to assess the potential need for primary prevention implantable cardioverter-defibrillator (ICD) implantation after complete revascularization and implementation of optimal medical therapy?	T	T
**Q33)** Which is incorrect in patients with Acute coronary syndrome with cancer?	F	F
**Q34)** Which is not correct about arrhythmias developing due to acute coronary syndrome?	T	F*
**Q35)** Which of the recommendations is not correct in cases of high-grade AV block without sinus bradycardia or stable escape rhythm with hemodynamic intolerance due to acute coronary syndrome?	T	F*
**Q36)** Which of the recommendations for myocardial infarction with non-obstructive coronary arteries is incorrect?	T	T

**Q37)** Which of the following approaches is incorrect regarding multivessel disease in hemodynamically stable STEMI patients undergoing PPCI?	T	T
**Q38)** According to acute coronary syndrome guidelines, which of the following is not among the recommendations on technical aspects of invasive strategies?	T	T
**Q39)** Which of the strategies recommended to reduce the risk of bleeding associated with percutaneous coronary intervention is incorrect?	T	T
**Q40)** Which of the following are contraindications for nitroglycerin (NTG) in patients with ACS and continuing chest pain?	T	T
**Q41)** For patients with ACS, what is the recommended duration of double antiplatelet therapy (DAPT)?	T	T
**Q42)** Absolute contraindications for thrombolytics include which of the following?	T	T
**Q43)** Relative contraindications for thrombolytics include which of the following?	F	F
**Q44)** Which option is not among the high-risk features of stent-induced recurrent ischemic events?	F	F
**Q45)** In patients with acute coronary syndromes without ST-segment elevation, which one is not found among the electrocardiographic abnormalities?	T	T
**Q46)** According to the Academic Research Consortium on High Bleeding Risk during percutaneous coronary intervention, which of the following is not among the major criteria for high bleeding risk?	T	T
**Q47)** According to the Academic Research Consortium on High Bleeding Risk during percutaneous coronary intervention, which of the following is not among the minor criteria for high bleeding risk?	F	T*
**Q48)** Which of the following is not typically a clinical presentation of acute coronary syndrome?	T	F*
**Q49)** After a large myocardial infarction, when does the troponin level peak?	F	T*
**Q50)** In acute STEMI, primary PCI is preferred over fibrinolysis in which of the following situations?	T	T

*: Questions initially answered incorrectly by ChatGPT-4 but subsequently corrected when asked again on the 30th day.
